# Evaluation of *Toxoplasma gondii* infection in western Iran: seroepidemiology and risk factors analysis

**DOI:** 10.1186/s41182-020-00222-x

**Published:** 2020-05-19

**Authors:** Morteza Mousavi-Hasanzadeh, Hossein Sarmadian, Reza Ghasemikhah, Mojtaba Didehdar, Maryam Shahdoust, Mahshid Maleki, Mahdieh Taheri

**Affiliations:** 1 Student Research Committee, Arak University of Medical Sciences, Arak, Iran; 2 Department of Infectious Diseases, Arak University of Medical Sciences, Arak, Iran; 3 Departments of Parasitology and Mycology, Arak University of Medical Sciences, Arak, Iran; 4 Department of Biostatistics, School of Public Health, Hamadan University of Medical Sciences, Hamadan, Iran; 5 Department of Medical, Faculty of Nursing and Midwifery, Isfahan University of Medical Sciences, Isfahan, Iran; 6 Department of Medical, Faculty of Nursing and Midwifery, Iran University of Medical Sciences, Tehran, Iran

**Keywords:** General population, Toxoplasmosis, IgG anti-*Toxoplasma*, *Toxoplasma gondii*

## Abstract

**Background:**

Toxoplasmosis is a parasitic infectious disease, and *Toxoplasma gondii* is the causative factor of this intracellular protozoan disease. Due to the lack of information about the rate of *T. gondii* in general papulation of Markazi Province in Iran, the current study was conducted to determine the prevalence of toxoplasmosis and the related risk factor analysis in the general population of Markazi Province.

**Methods:**

This cross-sectional study was performed within 6 months on individuals who were referred to diagnostic laboratories in Markazi Province. The demographic and background information of the subjects were collected using a questionnaire. Three milliliters of blood samples was collected from the participants under sterile conditions. The sera were separated and evaluated for levels of anti-*Toxoplasma* IgG antibody using a commercial enzyme-linked immunosorbent assay (ELISA) method. The collected data were analyzed by the SPSS software using descriptive statistics and chi-square test.

**Results:**

Out of 824 people from the general population of Markazi Province who were investigated in this study, 276 (33.5%) had anti-*Toxoplasma* antibodies in their blood. According to the logistic regression model, gender variables, location, marital status, and having a cat at home do not affect the chances of contracting the parasite. Furthermore, the chance of contracting the parasite in 41- to 50-year-olds is 0.85 times the one in the 20- to 30-year-olds. The prevalence of toxoplasmosis in men and women in Markazi Province was 33% and 34.5%, respectively.

**Conclusion:**

The mean prevalence of *T. gondii* infection in the age groups of 20-40, and ≥ 40 years was estimated to be 24.7%, and 40.8%, respectively. These rates were significantly lower than the national results (44%, and 55%, respectively). Therefore, regarding to the health authorities, it is necessary to raise the level of awareness of people of the region, especially at-risk groups about the transmittance and prevention methods, and infection risk factors in order to prevent the occurrence of *T. gondii* infection and reduce the prevalence and incidence of the disease.

## Introduction

*Toxoplasma* (*T.*) *gondii* is an intracellular protozoan with widespread global distribution and it is estimated that one-third of the world’s adult population to be infected with the parasite [1].

The infection can occur through consumption of the oocyst-contaminated food, water or raw meat products containing parasite cysts as well as mother-to-the fetus infection. The life cycle of the parasite becomes completed as it transfers from the warm-blooded intermediate hosts to the cat as the final host [2, 3].

Only 10–30% of new *toxoplasmosis* infections in humans cause clinical symptoms. Symptoms may range from subclinical lymphadenopathy to the fatal central nervous system disease as well as other pathologies in immunodeficient patients. In some congenital infections, some symptoms such as seizures and mental retardation may occur [4].

The seroprevalence of *T. gondii* infection is quite different among human communities which largely depend on the geography, climate, nutrition-related habits, and sanitation levels [5].

In Iran, most studies have been performed to determine the prevalence of *T. gondii* infection in high-risk groups such as pregnant women, premarital women, neonates, and children, and only few studies have been performed on the general population [6–12]. In this regard, Markazi Province is not an exception and as the other provinces in Iran, except of a few limited studies on specific populations, the prevalence of *T. gondii* infection on the general population of this province has not been studied [9].

Altogether, the objectives of this study are to determine the prevalence of *T. gondii* infection and also to analyze the risk factors in the general population of Markazi Province.

## Material and methods

### Ethics statement

This study was conducted after obtaining permission from the Ethics Committee of Arak University of Medical Sciences under the number of IR.ARAKMU.REC.1396.15. At the beginning of the study, a signed informed consent form was obtained from each of the under-study individuals.

### The study area

**Fig. 1:**
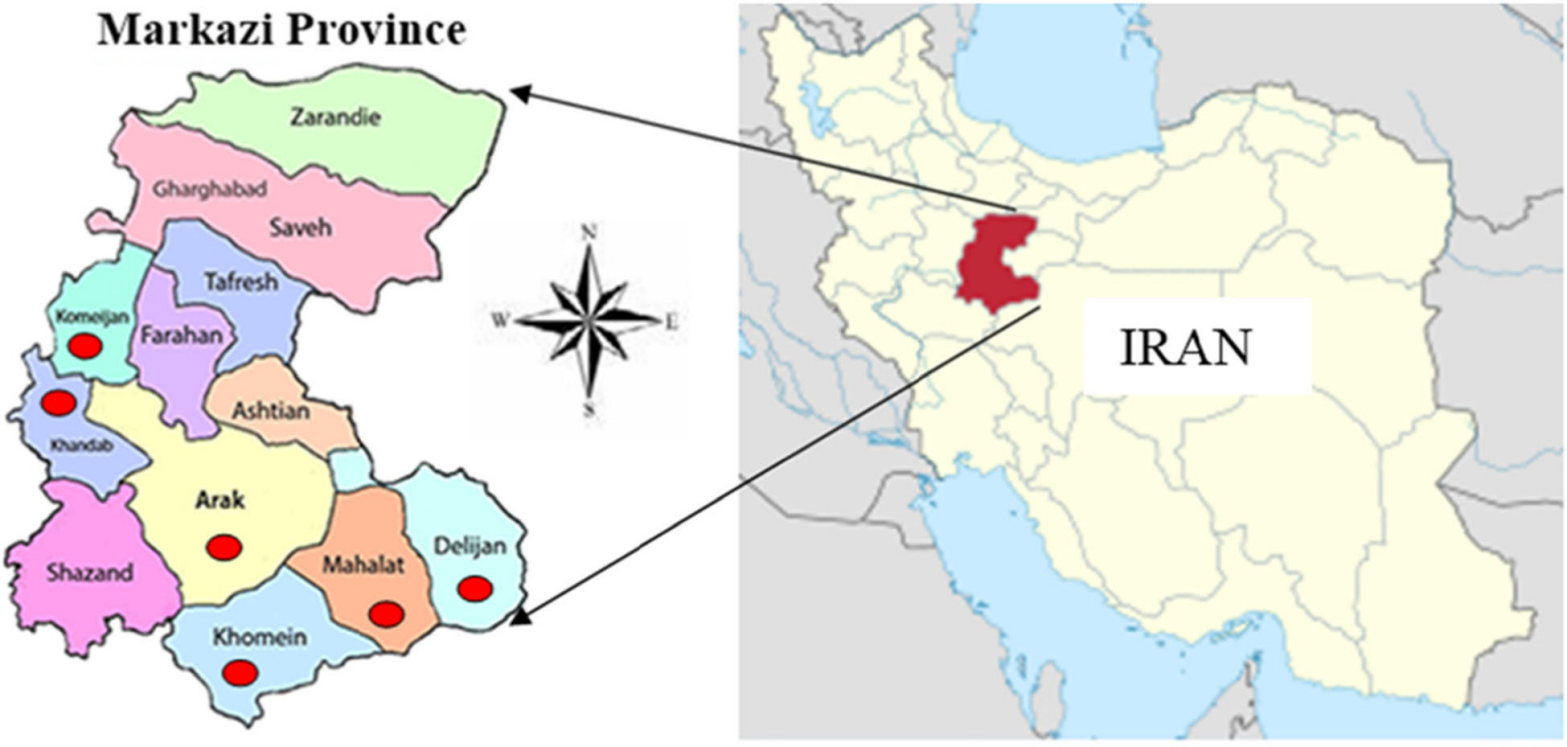
Location of Markazi Province in the present prevalence study

This cross-sectional study was carried out from May to October 2017, in six cities containing Arak, Khomain, Khondab, Mahallat, Komijan, Delijan, located in Markazi Province, western Iran (Fig. 1).

The study region has a variety of climates due to its high mountains and adjacent to the central border of the country. Gardening and animal husbandry are common in all mentioned areas except of Arak city which is an industrial area.

### Sampling and sociodemographic assessment

This cross-sectional study was performed within 6 months on individuals who referred to laboratories affiliated with Arak University of Medical Sciences, in Markazi Province. The number of samples was calculated based on the official population statistics of each city in this province. A questionnaire was used to collect demographic information including age, gender, marital status, and residence status as well as some risk factors of *T. gondii* including pregnancy and abortion history, and cat-care history. The 3 ml blood samples were collected from the patients and coded using disposable sterile syringes under safety conditions.

Blood samples were kept overnight at ambient temperature in order to allow clot formation and were centrifuged at 1000 g for 10 min at room temperature. The obtained serum samples were collected in a 2-ml Eppendorf tubes and were stored at 4 °C for 24 h until transportation in an icebox to Parasitological Laboratory, School of Medicine, Arak University of Medical Sciences, Markazi Province where samples were kept at −20 °C until analysis [13].

### Serological test

IgG anti-*T. gondii* antibody assay kit is designed with capture antibody method. Plate wells were coated with anti-human antibody. The levels of anti-*Toxoplasma* IgG antibodies were measured using a Pishtazteb kit according to the manufacturer’s instructions. The optical density (OD) of the samples was measured at 450 nm and 630 nm using a plate reader (BioTek; Winooski, Vermont, USA). Index value larger than 1.1 was considered as positive. A range between 0.9 and 1.1 was considered as equivocal, and index value less than 0.9 was considered as negative. Positive and negative serum controls were included in every plate.

### Statistical analysis

The SPSS software version 16 was used for statistical analysis. Data was presented as descriptive tables. The qualitative variables were also compared using the chi-square test. A *p* value smaller than or equal to the 0.05 was considered as significant. Logistic regression tests were used to determine the relations between the risk factors of *T. gondii infection* and their incidence rates.

## Results

In this study, 824 individuals were investigated. Table 1 shows the general characteristics of the studied population which their majority comprised urban married women aged 31-50 years.

**Table 1:** Overview on the demographic characteristics of the population under study

Percent (%)	Numbers	Variables
Sex		
65.2	537	Female
34.8	287	Male
Age groups (year)		
15.7	129	20-30
29.9	247	31-40
32.8	270	41-50
21.6	178	51-60
		Habitat
69.5	573	Urban
30.5	251	Rural
		Marital status
11.5	95	Single
88.5	729	Married
		Abortion history in women
17.1	92	Yes
82.9	445	No
		Pregnancy history of women
70.9	381	Yes
29.1	156	No
		Keeping cat
8.6	71	Yes
91.4	753	No

Table 2 illustrates the prevalence of *T. gondii* infection in the counties of Markazi Province. It indicates that this disease is most prevalent in Arak with a contamination rate of 21.6% (*p* < 0.05).

**Table 2:** The prevalence of asymptomatic toxoplasmosis in the city of the Markazi Province

City	Seropositive samples (%)	Seronegative samples (%)	Total
Arak	178 (21.6)	421 (51.2)	599 (72.8)
Khomain	24 (2.9)	25 (3)	49 (5.9)
Khondab	6 (0.73)	42 (5.1)	48 (5.8)
Mahallat	25 (3)	23 (2.8)	48 (5.8)
Komijan	15 (1.8)	17 (2.1)	32 (3.9)
Delijan	28 (3.4)	20 (2.4)	48 (5.8)
Total	276 (33.5)	548 (66.5)	824 (100)

The results of the serological test showed that out of 824 blood samples from Markazi Province, 33.5% had anti-*Toxoplasma* IgG antibodies (276 positive cases) and 66.5% did not have it (548 negative cases). Table 3 demonstrates the frequency of anti-*Toxoplasma* IgG antibodies in relation with the demographic characteristics of the studied population.

**Table 3:** Analysis of seroprevalence of toxoplasmosis based on some demographic characteristics of the population under study based on logistic regression

Variables	Seropositive samples (%)	Seronegative samples (%)	OR	*p value*
**Sex**				
Female	177 (33)	360 (67)	0.934	0.657
Male	99 (34.5)	188 (65.5)		
**Age groups (year)**				
20-30**	37 (28.7)	92 (71.3)	-	-
31-40	56 (22.8)	191 (77.2)	1.37	0.475
41-50	87 (32.2)	183 (67.8)	0.846	0.0001*
51-60	96 (53.9)	82 (46.1)	0.344	0.074
**Habitat**				
Urban	194 (33.9)	379 (66.1)	1.055	0.739
Rural	82 (32.7)	169 (67.3)		
**Marital status**				
Single	27 (28.4)	68 (71.6)	0.765	0.265
Married	249 (34.2)	480 (65.8)		
**Keeping cat**				
Yes	24 (33.8)	47 (66.2)	1.015	0.954
No	252 (33.5)	501 (66.5)		

*Statistical significance was defined as a *p* value of < 0.05

**The reference category

Table 3 represents the results of the adjusted regression model using gender variables including age (classified into four groups of 20-30 years, 31-40 years, 41-50 years, and 51-60 years), place of residence (city or village), marital status (single or married), and having a cat at home.

Based on the results, only the age group variable played a role. This means that the variables of sex, location, marital status, and having a cat do not affect the chances of contracting the *toxoplasma* parasite.

We should notice that the age variable between 41-50 years (*p* value < 0.05) remained notable in the model and it is not confirmed that the other age group coefficients have a remarkable level of 5%.

According to the findings, the chance of contracting the *toxoplasma* parasite in people with age between 41 and 50 years is 0.85 times than the one in people with age between 20 and 30 years.

Although, the regression coefficient corresponding to the people with age between 31 and 40 years does not play a role in the adjusted regression model, the chance of contracting the parasite in this group of people is 1.37 times higher than in people with age between 20 and 30 years.

Therefore, based on the results, the variables of sex, location, marital status, and having a cat do not affect the chances of contracting the *toxoplasma* parasite.

## Discussion

This is the first cross-sectional study on seroprevalence of *T. gondii* infection among the general population of Markazi Province in central Iran. The basis of this study was the measurement of IgG in the serum of the studied subjects by ELISA method. Due to the role of humoral immunity in immune responses and infection prevention, IgG antibody measurement can be evaluated as an indicator to assess the personal immunity to the infection. Therefore, using the enzymatic immunoassay methods such as ELISA kit in diagnosing the level of immunity can be helpful.

The results of the present study showed that the prevalence of asymptomatic infection was 33.5% in the general population of Markazi Province. The comparison of the current results with those of previous studies in the same region of Iran (Arak city) showed that there was an approximate similarity in the prevalence of this disease between 1026 males who referred to a pre-marriage laboratory (35.5%), 308 pregnant women (38%), and 261 postpartum women in a maternity hospital (32.5%). Contradictory, there was a sensible difference between the prevalence obtained in the present study and the serological prevalence of the disease in 400 girls of reproductive age (24.3%) and 49 HIV-positive patients (20.4%) in Arak city [7, 14].

A review of other sero-epidemiological studies of *T. gondii* in Iran showed that the prevalence of *T. gondii* infection in the general population of Markazi Province is consistent with the one in the general population of Iran. The 23,385 cases out of 52,294 people who were investigated in different regions of Iran from the year 1978 to 2012, to identify anti-*Toxoplasma* IgG, tested positive. The meta-analysis of the available data showed that the prevalence of asymptomatic infection is in the range of 33-45.7% (with an average of 33.3%) among the general population of Iran [8].

In a recent systematic review, Rostami et al. assessed the global prevalence of latent toxoplasmosis in pregnant women using published studies. They obtained a prevalence of 33.8% in this group of people. The countries with low income and low human development indices showed the highest prevalence [15]. In addition, in the published meta-analysis, the prevalence of *T. gondii* infection was assessed in immunocompromised individuals. Based on the results of this meta-analysis, the estimated pooled prevalence of toxoplasmosis in HIV-infected patients, cancer patients, and transplant recipients was reported as 42.1%, 26.0%, and 42.1%, respectively [16].

The serological prevalence of *T. gondii* infection in men and women in Markazi Province was 33% and 34.5%, respectively which is lower than what is reported as 42% in women and 44% in men in Iran [8]. It is noteworthy that in both provincial and national studies, the prevalence of asymptomatic is higher in men than in women. The meta-analysis of the available data showed that the prevalence of asymptomatic infection is in the range of 33-45.7% (with the average of 33.3%) among the general population of Iran [8].

The mean prevalence of *T. gondii* infection in the age groups of 20-40 and ≥ 40 years was estimated to be 24.7%, and 40.8%, respectively which was significantly lower than the national result (44%, and 55%, respectively) [8].

The prevalence of *T. gondii* infection in the general population of Markazi Province like the one in the general population of Iran is significantly higher in people who are in contact with cats [8].

The prevalence of *T. gondii* infection in the current study was compared with the one found in similar studies in some adjacent provinces. In several studies on the prevalence of this parasite in Tehran the highest and the lowest prevalence rates were 68.4% [12], and 33.9% [17], respectively with an average of 49% [8]. This significant difference in the prevalence of *T. gondii* infection in this city is due to the socioeconomic status of the population in different parts of this metropolis. The prevalence of this disease in Isfahan Province was reported in three studies as 41.4% [18], 29.26% [19], 51.25% [20], and 50.8% [21] with an average of 40% [8]. According to our results, the prevalence of *T. gondii* infection in Markazi Province is almost similar to the one in Tehran Province. The climatic similarity of these two provinces may also play a role in this prevalence rate similarity.

The comparison of the prevalence of *T*. *gondii* infection in Markazi Province with the other regions of the country showed that the prevalence of this disease in the province is very similar to the one in all climates except of the temperate and humid regions in northern Iran [11]. This result is not unexpected as Markazi Province has a diverse climates changing from mid-desert to cold mountainous [22].

Studeničová et al. reported a seroprevalence of *T. gondii* infection antibodies among 508 healthy individuals in Slovakia. Using the ELISA method for diagnosis, the prevalence of IgG antibody in the studied population was estimated to be 24.2% (123 out of 508 cases). This study also showed that the prevalence of *T*. *gondii* infection would increase significantly with age increasing. However, the prevalence of *T. gondii* infection in men and women as well as in rural and urban areas did not differ significantly [23].

Another study reported a high prevalence of IgG antibody in the inhabitants of Jakarta. The results indicated that the seroprevalence rate was 70% and there was no significant difference between men and women in the prevalence of *T. gondii* infection [24]. Alvarado-Esquivel et al. in 2011 reported that the prevalence of IgG anti–*T. gondii* antibody in 974 inhabitants in Durango City in Mexico using the ELISA method was 6.1% [25].

Another study using ELISA method in the rural area Okcheon-gun in Korea showed that the prevalence of *T*. *gondii* infection in women and men in the seventies or higher was significantly higher than in other groups. In accordance with the results of this study, we observed a correlation between increasing the prevalence of antibodies against *T. gondii* and age increasing. While our study showed a higher prevalence of *T. gondii* infection in women, this study did not show a significant difference in men (6.0%) and women (7.2%) [26].

In this study, we found that among all the investigated risk factors, only age had a significant effect on the outcome of contracting the parasite. In a study by Li et al., three variables including keeping cats at home, consuming uncooked meats, and the increasingly eating fresh oysters in recent years, were regarded to be risk factors for developing *T. gondii* infection in diabetic patients. Consumption of fresh oysters is common in recent years [27]. Nissapatorn et al. reported no significant relationship between the prevalence of toxoplasmosis and the related risk factors in expecting women [28]. Achaw et al. Remm reported that living in the city hand having minimal secondary education levels as major risk factors in *T. gondii* infection among pregnant women under prenatal care in downtown Babo Diolasso [29]. Adhroey et al. found that drinking untreated water to be a significant risk factor for higher *T. gondii* seroprevalence in China [30].

Our study has several limitations. Firstly, although both IgG and IgM antibodies are commonly studied in seroepidemiology of *T*. *gondii* studies, in this study the IgM measurement was excluded for three reasons as follows: (1) financial resource constraints, (2) the aim of the study which was to estimate the epidemiology of *T*. *gondii* in Markazi Province and not to diagnose and treat it, (3) detection of IgG but not IgM against *T. gondii* infection defines the classical serologic pattern of latent infection. In other word, IgG antibodies are used in epidemiological studies of chronic infectious diseases. Secondly, data analyzed in Markazi Province cannot be generalized to all provinces of the country. As these data are not real representations of the entire country of Iran, they cannot be compared to the results of national studies. Lastly, in order to compare the results with those obtained from other countries, data from a larger and more reliable statistical population in Iran should be collected.

## Conclusion

To our knowledge this is the largest study of the seroepidemiology of *T. gondii* in the general population of the Markazi Province. Given the 33.5% prevalence of asymptomatic infection in the general population of Markazi Province and regarding to the health authorities, it is necessary to raise the level of awareness of the people of the region, especially at-risk groups about the transmittance and prevention methods, and infection risk factors in order to prevent the occurrence of *toxoplasma* and reduce the prevalence and incidence of the disease.

## Data Availability

Input data for the analyses are available from the corresponding author on request.

## Abbreviations

ELISA: Enzyme-linked immunosorbent assay
IgG: Immunoglobulin Gamma
IgM: Immunoglobulin M
HIV: Human immunodeficiency viruses
